# 非小细胞肺癌PI3Kp110β高表达的临床意义及机制

**DOI:** 10.3779/j.issn.1009-3419.2017.12.03

**Published:** 2017-12-20

**Authors:** 焰 熊, 琳琳 曲, 东 李, 颖 王, 挺 李

**Affiliations:** 100034 北京，北京大学第一医院病理科 Department of Pathology, Peking University First Hospital, Beijing 100034, China

**Keywords:** 肺肿瘤, PI3Kp110β, 高表达, 临床意义, 机制, Lung neoplasms, PI3Kp110β, Overexpression, Clinical significance, Mechanism

## Abstract

**背景与目的:**

磷脂酰肌醇-3激酶（phosphatidylinositide 3-kinases, PI3K）通路是细胞内最重要的信号传导通路之一，参与细胞生长、增殖、分化、运动等多项重要功能，其异常活化见于50%-70%的非小细胞肺癌（non-small cell lung cancer, NSCLC）。PI3K蛋白作为PI3K通路的枢纽，表达水平的变化直接影响通路的功能状态，与NSCLC的发生、发展和靶向治疗密切相关。本研究旨在探讨PI3K催化亚基p110β在NSCLC中的表达、临床意义及作用机制。

**方法:**

采用免疫组化法检测170例NSCLC中p110β和PI3K通路其他蛋白的表达，分析p110β的表达与患者临床病理特征的关系，与PI3K通路其他蛋白表达的关系。

**结果:**

p110β在NSCLC的高表达率为41.8%。其表达水平与Ki67计数呈正相关（*P*=0.040），在腺癌和鳞状细胞癌间、不同性别、年龄、吸烟状况分组间以及不同T分期、N分期、TNM分期和病理学分级间差异均无统计学意义（*P*＞0.05）。p110β的高表达与EGFR突变呈负相关（*P*=0.022），与PTEN表达缺失呈正相关（*P*＜0.001），与ROS1、野生型EGFR、HER2、ALK和p-Akt（Ser473）的表达均无明显关系（*P*＞0.05）。

**结论:**

p110β高表达在NSCLC是一频发事件；与PTEN缺失密切相关，是PTEN缺失肿瘤生存和进展的关键；可导致肿瘤增殖指数升高，但却与磷酸化Akt无关。*PIK3CB*突变不是p110β高表达的主要原因；各类受体酪氨酸激酶（receptor tyrosine kinases, RTKs）异常均未显示导致癌组织p110β表达增高的能力，而且，*EGFR*突变者p110β的表达反而低于EGFR野生型。

## Abstract (en)

**Background and objective:**

Phosphatidylinositide 3-kinases (PI3K) pathway is one of the most important pathway in cells, which plays an important role in proliferation, growth, differentiation and mobility of cells. The aberrant activation of PI3K pathway was exsited in 50%-70% cases of non-small cell lung cancer (NSCLC). As the key point in PI3K pathway, expression of PI3K plays a critical role in activity of the pathway, which is closely related with the initiation and development of NSCLC, furthermore with the response of tumor to target treatment. Our study is to analyze the clinical significance and mechanism of PI3K p110β overexpression in NSCLC.

**Methods:**

Expression of p110β and other proteins in PI3K pathway were detected by immunohistochemistry in 170 cases of NSCLC. Correlation between expression of p110β and clinicopathological characteristics of patients as well as expression of other proteins in PI3K pathway was analyzed.

**Results:**

In 170 NSCLC, overexpression of p110β was found in 41.8% of cases. Correlation between overexpression of p110β and Ki 67 index was significant (*P*=0.040). No significant difference of p110 expression were observed among different cohorts of gender, age, smoking status, classification, grade and stage (*P* > 0.05). Correlation between expression of p110β and other proteins in PI3K pathway was various, positively correlated with PTEN loss (*P* < 0.001) and negatively correlated with mutant *EGFR* (*P*=0.022), while not correlated with P-Akt (Ser473), HER2, ALK, ROS1 and wild type *EGFR* (*P* > 0.05).

**Conclusion:**

Overexpression of p110β is frequently detected in NSCLC. It is closely related with PTEN loss NSCLC, which shows that it plays an important role in maintaining and developing of tumors driven by PTEN loss. It initiates the proliferation of tumor cells in NSCLC without phosphorylating Akt. *PIK3CB* mutation is not the major cause of overexpression of p110β. Dysregulation of receptor tyrosine kinases (RTKs) doesn't show potential of increasing p110β level in cancer tissue, furthermore the expression of p110β in tumors with *EGFR* mutation is lower than in tumors without *EGFR* mutation.

肺癌的发病率和死亡率在全球范围内均居恶性肿瘤首位。2012年，中国肺癌新发病例70.48万例，发病率为52.06/10万，占全部新发恶性肿瘤的19.65%；死亡病例56.94万例，死亡率为42.05/10万，占全部恶性肿瘤死亡人数的26.04%，其中，非小细胞肺癌（non-small cell lung cancer, NSCLC）占全部肺癌病例的75%-85%^[1]^。

磷脂酰肌醇-3激酶（phosphatidylinositide 3-kinase, PI3K）通路是细胞内最重要的信号传导通路之一，参与细胞生长、增殖、分化、运动等多项重要功能，其异常活化见于多种肿瘤^[2]^，在NSCLC的异常活化率高达50%-70%^[3]^，成为肺癌研究和治疗的热点^[4]^。PI3K蛋白作为PI3K通路的枢纽，其基因和蛋白水平的变化直接影响通路的功能状态，与肺癌的发生、发展、预后以及多种受体酪氨酸激酶（receptor tyrosine kinases, RTKs）抑制剂和PI3K抑制剂的疗效密切相关^[2]^。因此，针对PI3K广泛而深入的研究对评估肺癌患者预后、预测靶向治疗反应、研究耐药机制均具有重要意义。

PI3K分为Class Ⅰ、Class Ⅱ和Class Ⅲ三类，Class Ⅰ又进一步分为Class ⅠA和Class ⅠB两亚类。其中与肿瘤关系最密切的是Class ⅠA。Class ⅠA为异源二聚体，由一个催化亚基和一个调节亚基组成，催化亚基共有p110α、p110β和p110δ三种，调节亚基共有p85α、p85β、p55γ三种。催化亚基是PI3K的效应器，其表达水平与PI3K的功能状态密切相关。生理情况下，p110δ只在白细胞中表达，与淋巴造血系统肿瘤相关，p110α和p110β则在各种组织中广泛表达，与多种实体肿瘤相关^[5]^。迄今为止，有关肺癌中p110α表达的研究非常有限，p110β表达的研究才刚刚起步。本课题组在检测170例NSCLC组织p110β及PI3K通路其它蛋白表达的基础上，研究p110β高表达与临床病理特点的关系，在肺癌发生、发展中的作用，分析可能的机制和对信号通路的影响，进而为预测NSCLC的预后，为靶向治疗筛选患者，评估耐药性提供理论参考。

## 1 资料与方法

### 1.1 临床及病理资料

收集2015年1月-2015年12月北京大学第一医院经手术切除病理证实为腺癌和鳞状细胞癌的病例，共170例，程序符合北京大学第一医院临床研究伦理委员会所制定的伦理学标准（伦理审查批件号：2016[1111]）。患者根据年龄分为四组：≤44岁、45岁-59岁、60岁-74岁、75岁-89岁、90岁以上。吸烟状况分为两类：终身吸烟少于100支者为不吸烟；其他为吸烟^[6]^。肿瘤分期遵照美国癌症联合会（American Joint Committee on Cancer, AJCC）肿瘤分期手册（第八版）的标准^[7]^。病理学分级：鳞状细胞癌，肿瘤组织内可见大量角化珠及明显的细胞间桥为1级；肿瘤缺乏角化珠，仅见少许隐约可辨的细胞间桥为3级；介于1级和3级之间为2级。腺癌，贴壁型为1级，腺泡型/乳头型为2级，实体型/微乳头型为3级^[8]^。

### 1.2 标本处理

标本处理严格遵照2014年美国病理医师协会（College of American Pathologists, CAP）肺癌标本规范化处理程序：手术标本离体后即送病理科，由胸肺亚专科病理医生行大体检查；测量肿瘤体积，将10%中性缓冲福马林固定液沿大气道注入后，将整个标本浸泡于10倍以上体积的固定液；固定时间6 h-48 h内。固定后标本规范取材，石蜡包埋，4 μm切片。

### 1.3 免疫组织化学染色试剂与方法

采用抗体包括p110β（ab151549, 1:200, Abcam）、EGFR（UMAB95, 1:200, ORIGENE）、EGFR（L858R）（43B2, 1:200, Cell Signaling）、EGFR（E746-A750）（D6B6, 1:200, Cell Signaling）、HER2（4B5，工作液，Ventana）、MET（D1C2, 1:200, Cell Signaling）、ROS1（D4D6, 1:200, Cell Signaling）、ALK（D5F3, 1:200, Cell Signaling）、p-Akt（Ser473）（D9E, 1:100, Cell Signaling）、Ki67（MIB1, 1:150, Dako）、PTEN（6H2.1, 1:100, Dako）。抗原修复应用pH 9.0的乙二胺四乙酸缓冲液，温度98 ℃，时间为20 min，由EnVision^TM^ FLEX+Rabbit Linker（Dako公司）放大信号，采用EnVision^TM^ FLEX/HRPD Detection System（Dako公司）检测信号，由Autostainerlink 48全自动免疫组化机（Dako公司）完成。

### 1.4 免疫组织化学染色结果判定

特异阳性信号的亚细胞定位：Ki67为细胞核；ROS1、ALK和PTEN为细胞浆；HER2、MET、EGFR、EGFR（L858R）和EGFR（E746-A750）为细胞膜、细胞浆、细胞膜/浆；p110β和p-Akt（Ser473）为细胞核、细胞浆、细胞核/浆。

p110β、HER2、EGFR采用如下标准：中-强着色肿瘤细胞比率＞10%为高表达，中-强着色肿瘤细胞比率≤10%为低表达（图 1）。MET采用如下标准：中-强着色肿瘤细胞比率≥50%为高表达，中-强着色肿瘤细胞比率＜50%为低表达^[9]^。p-Akt（Ser473）采用如下标准：任何强度着色的肿瘤细胞比率＞10%为阳性，肿瘤细胞不着色或着色细胞比率≤10%为阴性。EGFR（L858R）和EGFR（E746-A750）采用如下标准：中-强着色肿瘤细胞比率＞10%为阳性，中-强着色肿瘤细胞比率≤10%为阴性；EGFR（L858R）和/或EGFR（E746-A750）阳性总称为突变*EGFR*阳性，EGFR（L858R）和EGFR（E746-A750）均阴性总称为突变*EGFR*阴性^[10]^。ALK采用如下标准：肿瘤细胞浆中存在强颗粒状染色（任何阳性细胞百分比）为阳性，肿瘤细胞浆中无强颗粒状染色为阴性^[11]^。ROS1采用国际肺癌研究协会（International Association for Study of Lung Cancer, IASLC）推荐标准：肿瘤细胞弥漫均匀着色无论强度如何均为阳性，肿瘤细胞均不着色或着色细胞斑片状分布无论强度如何均为阴性。PTEN采用如下标准：肿瘤细胞无着色（周围正常组织着色）为表达缺失，任何比率的肿瘤细胞任何强度着色为有表达^[12]^。Ki67的计数方法：于阳性信号最密集区，计数2, 000个肿瘤细胞，以核阳性细胞所占百分数为Ki67指数^[13]^。Ki67计数分组标准：≤10%；＞10%且≤30%；＞30%且≤50%；＞50%。

**1:**
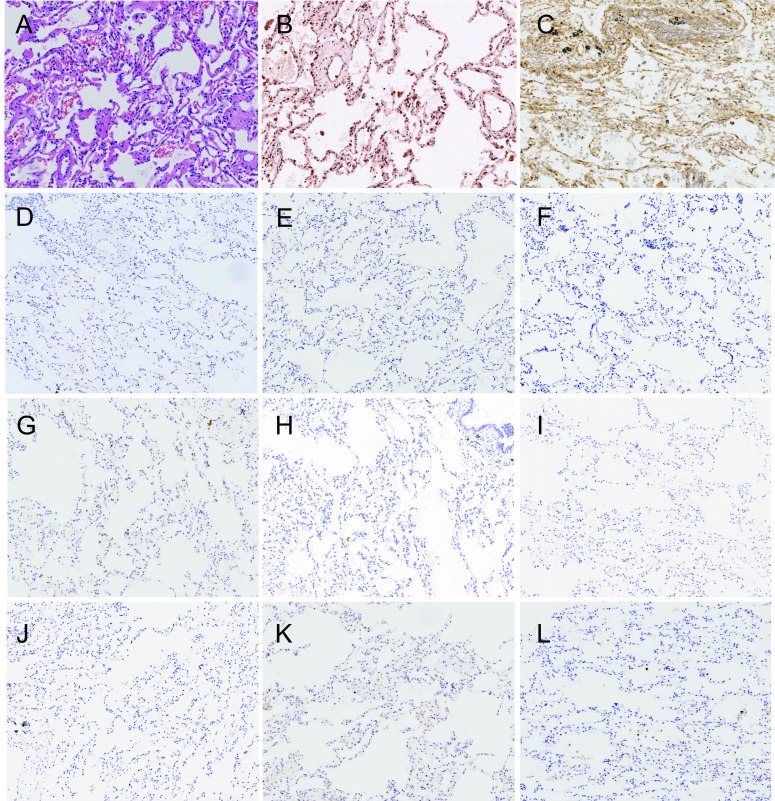
正常肺组织、组织形态和PI3K通路蛋白表达（×100）。A：组织形态（HE）；B：p110*β*低表达（IHC）；C：PTEN正常表达（IHC）；D：EGFR（L858R）阴性（IHC）；E：EGFR（E746-A750）阴性（IHC）；F：ALK（D5F3）阴性（IHC)；G：MET低表达（IHC）；H：ROS1阴性（IHC）；I：EGFR低表达（IHC）；J：HER2低表达（IHC)；K：p-Akt（Ser473）阴性（IHC)；L：Ki67计数小于10%（IHC）。 Normal lung tissue, the morphology and expression of proteins in PI3K pathway (×100). A: Histological morphology (HE); B: Expression of p110*β* is low (IHC); C: Expression of PTEN is normal (IHC); D: Expression of EGFR (L858R) is negative (IHC); E: Expression of EGFR (E746-A750) is negative (IHC); F: Expression of ALK (D5F3) is negative (IHC); G: Expression of MET is low (IHC); H: Expression of ROS1 is negative (IHC); I: Expression of EGFR is low (IHC); J: Expression of HER2 is low (IHC); K: Expression of p-Akt (Ser473) is negative (IHC); L: Ki67 index is less than 10% (IHC).

### 1.5 统计学分析

采用SPSS 21.0统计软件包对实验数据进行统计学分析处理。分析p110β在各临床病理分组间表达的差异以及与PI3K通路其他蛋白表达的相关性均采用卡方检验。以*P*＜0.05为差异有统计学意义。

## 2 结果

### 2.1 临床病理特征

170例NSCLC，男性108例（63.5%），女性62例（36.5%）；中位年龄63.00岁；吸烟者93例（54.7%），不吸烟者77例（45.3%）；腺癌95例（55.9%），鳞状细胞癌75例（44.1%）（表 1）；早期（Ⅰ期和Ⅱ期）患者为主，共147例（86.5%）；病理学分级以低级别为主，共131例（77.0%）；Ki67中位计数为25.9%，≤10%者占49.4%，＞10%且≤30%者占14.7%，＞30%且≤50%者占17.6%，＞50%者占18.3%（图 1-图 4）。

**1:** 170例非小细胞肺癌患者吸烟状况在病理分类、性别分组间的分布 The smoking status in different classification and gender cohorts of 170 patients of non-small cell lung cancer

Classification	Smoking		Total
	No	Yes	
Male			
SCC	9	60	69
AC	12	27	39
Female			
SCC	3	3	6
AC	53	3	56
Total	77	93	170
SCC: squamous cell carcinoma; AC: adenocarcinoma.			

**2:**
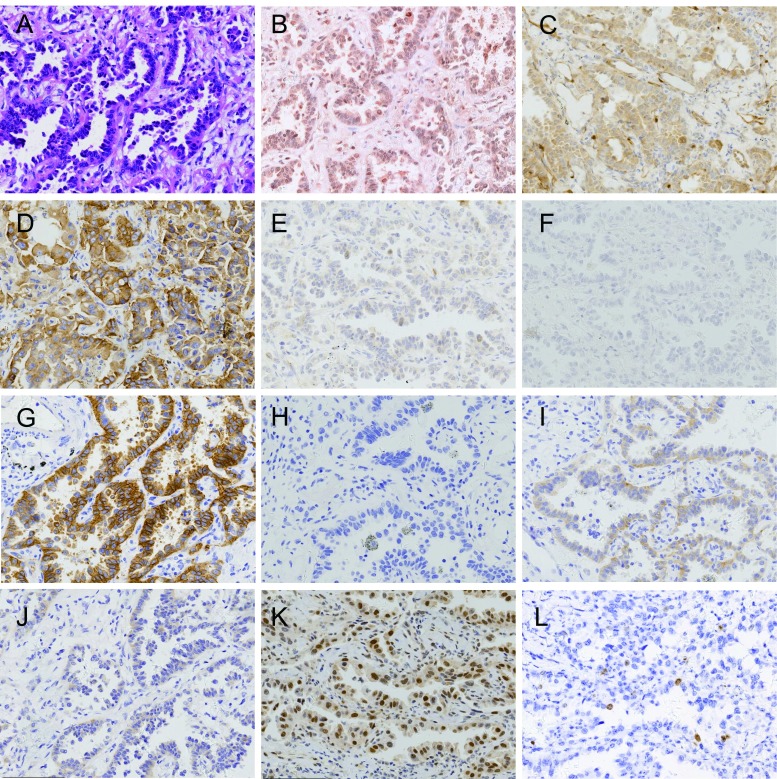
肺腺癌病例1、组织形态和PI3K通路蛋白表达（×200）。A：组织形态（HE）；B：p110*β*低表达（IHC）；C：PTEN正常表达（IHC）；D：EGFR（L858R）阳性（IHC）；E：EGFR（E746-A750）阴性（IHC）；F：ALK（D5F3）阴性（IHC）；G：MET高表达（IHC）；H：ROS1阴性（IHC）；I：EGFR低表达（IHC）；J：HER2低表达（IHC）；K：p-Akt（Ser473）阳性（IHC）；L：Ki67计数小于10%（IHC）。 Lung adenocarcinoma case 1, the morphology and expression of proteins in PI3K pathway (×200). A: Histological morphology (HE); B: Expression of p110*β* is low (IHC); C: Expression of PTEN is normal (IHC); D: Expression of EGFR (L858R) is positive (IHC); E: Expression of EGFR (E746-A750) is negative (IHC); F: Expression of ALK (D5F3) is negative (IHC); G: Expression of MET is high (IHC); H: Expression of ROS1 is negative (IHC); I: Expression of EGFR is low (IHC); J: Expression of HER2 is low (IHC); K: Expression of p-Akt (Ser473) is positive (IHC); L: Ki67 index is less than 10% (IHC).

**3:**
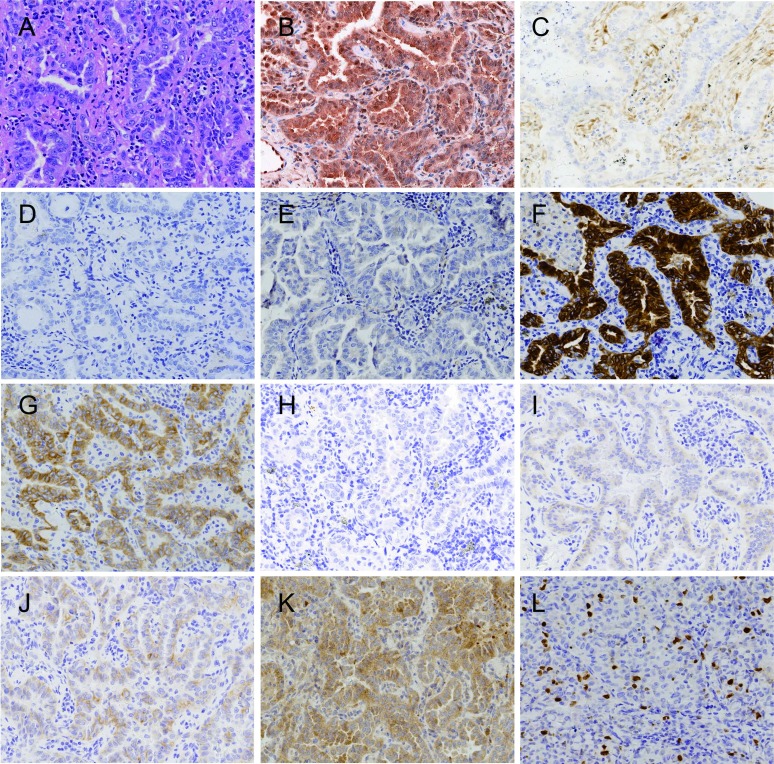
肺腺癌病例2组织形态和PI3K通路蛋白表达（×200）。A：组织形态（HE）；B：p110*β*高表达（IHC）；C：PTEN失表达（IHC）；D：EGFR（L858R）阴性（IHC）；E：EGFR（E746-A750）阴性（IHC）；F：ALK（D5F3）阳性（IHC）；G：MET高表达（IHC）；H：ROS1阴性（IHC）；I：EGFR低表达（IHC）；J：HER2低表达(IHC)；K：p-Akt（Ser473）阳性（IHC）；L：Ki67计数大于10%小于30%（IHC）。 Lung adenocarcinoma case 2, the morphology and expression of proteins in PI3K pathway (×200). A: Histological morphology (HE); B: Expression of p110*β* is high (IHC); C: Expression of PTEN is loss (IHC); D: Expression of EGFR (L858R) is negative (IHC); E: Expression of EGFR (E746-A750) is negative (IHC); F: Expression of ALK (D5F3) is positive (IHC); G: Expression of MET is high (IHC); H: Expression of ROS1 is negative (IHC); I: Expression of EGFR is low (IHC); J: Expression of HER2 is low (IHC); K: Expression of p-Akt(Ser473)is positive (IHC); L: Ki67 index is more than 10% and less than 30% (IHC).

**4:**
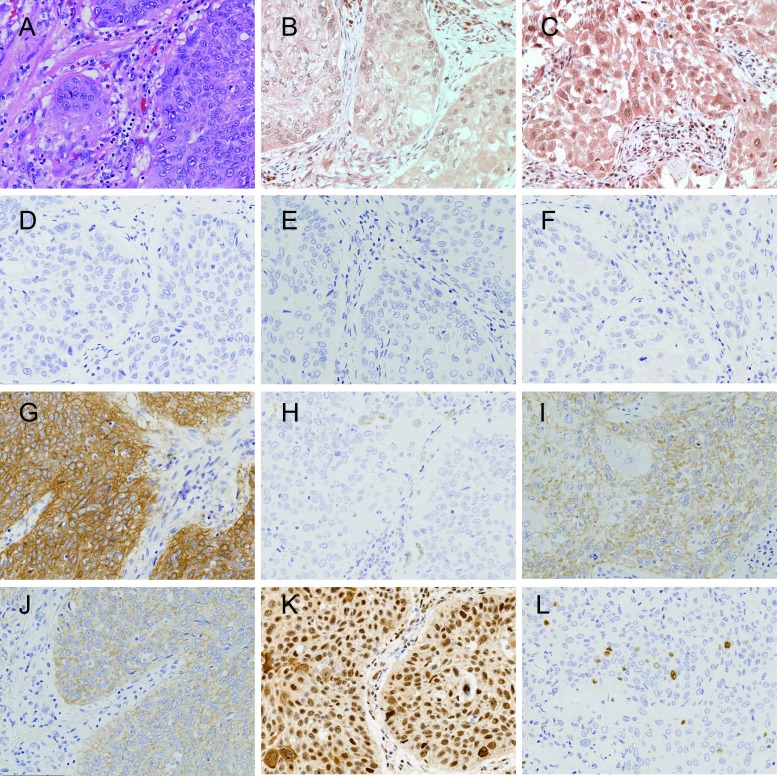
肺鳞状细胞癌病例1组织形态和PI3K通路蛋白表达（×200）。A：组织形态（HE）；B：p110*β*低表达（IHC）；C：PTEN正常表达（IHC）；D：EGFR（L858R）阴性（IHC）；E：EGFR（E746-A750）阴性（IHC）；F：ALK（D5F3）阴性（IHC）；G：MET高表达（IHC）；H：ROS1阴性（IHC）；I：EGFR低表达（IHC）；J：HER2低表达（IHC）；K：p-Akt（Ser473）阳性（IHC）；L：Ki67计数小于10%（IHC）。 Lung squamous cell carcinoma case 1, the morphology and expression of proteins in PI3K pathway (×200). A: Histological morphology (HE); B: Expression of p110*β* is low (IHC); C: Expression of PTEN is normal (IHC); D: Expression of EGFR (L858R) is negative (IHC); E: Expression of EGFR (E746-A750) is negative (IHC); F: Expression of ALK (D5F3) is negative (IHC); G: Expression of MET is high (IHC); H: Expression of ROS1 is negative (IHC); I: Expression of EGFR is low (IHC); J: Expression of HER2 is low (IHC); K: Expression of p-Akt (Ser473) is positive (IHC); L: Ki67 index is less than 10% (IHC).

### 2.2 p110β的表达及其在不同临床病理分组间的差异

170例NSCLC，p110β的高表达率为41.8%。p110β的表达与Ki67计数呈正相关（*P*=0.040）；在腺癌和鳞状细胞癌间，不同性别、年龄、吸烟状况分组间以及不同T分期、N分期、TNM分期和病理学分级间差异均无统计学意义（*P*＞0.05）（表 2）。

**2:** p110*β*在不同临床病理分组间表达的差异 Expression of p110*β* in different clinical pathological cohorts

Factors	*n*	p110*β*		*χ*^2^	*P*
		Low	High		
Gender				0.083	0.773
Male	108	62 (57.4%)	46 (42.6%)		
Female	62	37 (59.7%)	25 (40.3%)		
Age (yr)				0.910	0.823
≤44	5	2 (40.0%)	3 (60.0%)		
45-59	49	30 (61.2%)	19 (38.8%)		
60-74	101	58 (57.4%)	43 (42.6%)		
75-89	15	9 (60.0%)	6 (40.0%)		
Smoking				0.131	0.717
No	77	46 (59.7%)	31 (40.3%)		
Yes	93	53 (57.0%)	40 (43.0%)		
Classification				1.326	0.250
SCC	75	40 (53.3%)	35 (46.7%)		
AC	95	59 (62.1%)	36 (37.9%)		
T				0.830	0.842
T1	90	53 (58.9%)	37 (41.1%)		
T2	61	36 (59.0%)	25 (41.0%)		
T3	8	5 (62.5%)	3 (37.5%)		
T4	11	5 (45.5%)	6 (54.5%)		
N				1.479	0.477
N0	121	74 (61.2%)	47 (38.8%)		
N1	39	20 (51.3%)	19 (48.7%)		
N2	10	5 (50.0%)	5 (50.0%)		
M					
M0	170	99 (58.2%)	71 (41.8%)		
TNM				1.286	0.526
Ⅰ	105	62 (59.0%)	43 (41.0%)		
Ⅱ	42	26 (61.9%)	16 (38.1%)		
Ⅲ	23	11 (47.8%)	12 (52.2%)		
Grade				1.711	0.425
1	32	19 (59.4%)	13 (40.6%)		
2	99	54 (54.5%)	45 (45.5%)		
3	39	26 (66.7%)	13 (33.3%)		
Ki-67 (%)				8.335	0.040
≤10	84	54(64.3%)	30 (35.7%)		
＞10-30	25	9 (36.0%)	16 (64.0%)		
＞30-50	30	15 (50.0%)	15 (50.0%)		
＞50	31	21 (67.7%)	10 (32.3%)		
T: tumor; N: node; M: metastasis; TNM: tumor-node-metastasis.					

### 2.3 PI3K通路其他蛋白的表达及其与p110β表达的相关性

170例NSCLC，ALK阳性率为5.3%，ROS1阳性率为1.8%，突变EGFR阳性率为21.2%，PTEN缺失率为43.5%，野生型EGFR高表达率为31.8%，MET高表达率为40.0%，HER2高表达率为28.2%，p-Akt（Ser473）阳性率为49.4%。p110β的表达与EGFR突变呈负相关（*P*=0.022），与PTEN表达缺失呈正相关（*P*＜0.001）；与ALK、ROS1、野生型EGFR、HER2、和p-Akt（Ser473）的表达均不相关（*P*＞0.05）（表 3）。

**3:** p110*β*与PI3K通路其他蛋白表达的相关性 Correlation between expression of p110*β* and other proteins in PI3K pathway

Factors	*n*	p110*β*		*χ*^2^	*P*
		Low	High		
MET				1.951	0.162
Low	102	55 (53.9%)	47 (46.1%)		
High	68	44 (65.0%)	24 (35.0%)		
HER-2				0.108	0.742
Low	122	72 (59.0%)	50 (41.0%)		
High	48	27 (56.0%)	21 (44.0%)		
Wild type *EGFR*				0.022	0.881
Low	116	68 (58.6%)	48 (41.4%)		
High	54	31 (57.4%)	23 (42.6%)		
ROS1				2.190	0.139
Negative	167	96 (57.5%)	71 (42.5%)		
Positive	3	3 (100.0%)	0 (0.0%)		
ALK				0.743	0.389
Negative	161	95 (59.0%)	66 (41.0%)		
Positive	9	4 (44.4%)	5 (55.6%)		
Mutant *EGFR*				5.278	0.022
Negative	134	72 (53.7%)	62 (46.3%）		
Positive	36	27 (75.0%)	9 (25.0%)		
PTEN loss				83.284	0.000
Positive	96	85 (88.5%)	11 (11.5%)		
Negative	74	14 (18.9%)	60 (81.1%)		
p-Akt (Ser473)				0.113	0.736
Negative	86	49 (57.0%)	37 (43.0%)		
Positive	84	50 (59.5%)	34 (40.5%)		
MET: tyrosine-protein kinase Met; HER2: human epidermal growth factor receptor 2; EGFR: epidermal growth factor receptor; ROS1: proto-oncogene tyrosine-protein kinase ROS; ALK: anaplastic lymphoma kinase; PTEN: phosphatase and tensin homolog; p-Akt (Ser473): phosphorylate-Akt (Ser473).					

## 3 讨论

生理状态下，PI3K通路的模式为：细胞膜上的激酶蛋白活化，与PI3K调节亚基p85相结合，磷酸化p85为p-p85，进而将催化亚基p110募集到细胞膜内侧，p85解除对p110的抑制作用，PI3K获得活性，进而磷酸化磷脂酰肌醇4, 5-二磷酸盐（PIP2）产生磷脂酰肌醇3, 4, 5-三磷酸盐（PIP3），该通路中另一重要蛋白张力蛋白同源物（phosphatase and tensin homolog, PTEN）则通过去磷酸化PIP3为PIP2，拮抗PI3K的作用，是PI3K通路中重要的负性调节因子，在PI3K作用占优势的情况下，细胞内PIP3的浓度增加，与Akt结合，将Akt募集到细胞膜内侧并改变其构型，在同样位于细胞膜内侧的磷酸肌醇依赖激酶1（PDK1）的作用下，磷酸化成p-Akt获得完全活性，p-Akt转位到胞浆或胞核内磷酸化一系列底物，促进细胞增殖、抑制凋亡，因此，高表达p-Akt常被视为Akt异常活化的标志，进而提示PI3K通路的异常活化^[14]^。

编码p110β的基因*PIK3CB*位于3q22.3，长度为10 kb，迄今为止仅在乳腺癌细胞系HEK293T中检测到点突变E633K和D1067Y^[15, 16]^，在20%弥漫大B细胞淋巴瘤^[17]^和5%的乳腺癌^[18]^中检测到扩增，而在肺癌尚无基因突变报道，与基因突变的低频相比，肺癌p110β高表达则是一个高频事件，Cumberbatch等^[19]^报道为69.2%，本研究为41.8%，由此可见，肺癌p110β高表达的主要机制并非在基因水平。蛋白高表达的原因除了基因突变，还涉及mRNA转录、蛋白质翻译以及信号通路上游因子作用等多个层面。具体到PI3K的各亚基，由于PI3K通路在肿瘤中异常活跃，上游因子的调控是其高表达最主要的原因。虽然p110α和p110β的化学结构域相同，但其上游调节因子却不同，细胞系的研究显示p110α主要被磷酸化的RTKs活化，p110β主要被G蛋白偶联受体（G protein-coupled receptors, GPCRs）活化^[20]^。RTKs是一个跨膜激酶家族，包括酪氨酸蛋白激酶Met（tyrosine-protein kinase Met, MET）、人表皮生长因子受体2（human epidermal growth factor receptor 2, HER2）、表皮生长因子（epidermal growth factor receptor, EGFR）、原癌基因酪氨酸蛋白激酶ROS（proto-oncogene tyrosine-protein kinase ROS, ROS1）、间变淋巴瘤激酶（anaplastic lymphoma kinase, ALK）。本课题组既往的研究^[21]^显示肺癌p110α高表达与*EGFR*突变和MET高表达呈正相关，本研究显示p110β高表达与*EGFR*突变呈负相关，在组织学水平证实了RTKs对p110α和p110β的作用是不同的，与上述理论相符。至于如何解释*EGFR*突变的肺癌p110β表达低，迄今尚无类似文献报道，具体机制更不清楚，推测可能与*EGFR*突变刺激p110α高表达有关。p110α和p110β同为PI3K重要的催化亚基，在细胞内的表达可能存在一个此消彼长的动态平衡，在p110α高表达的情况下p110β被继发性地抑制。

**5:**
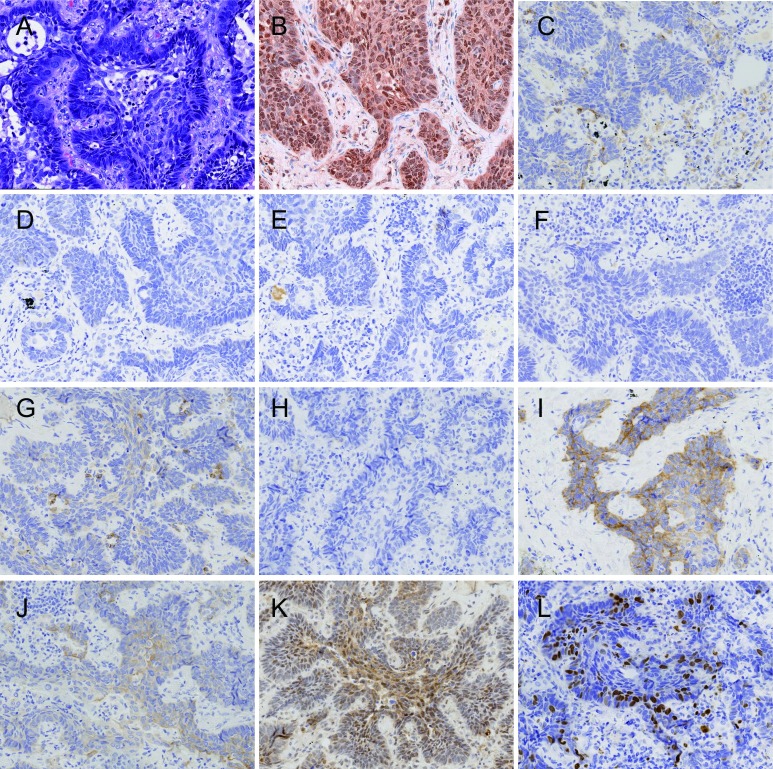
肺鳞状细胞癌病例2，组织形态和PI3K通路蛋白表达（×200）。A：组织形态（HE）；B：p110*β*高表达（IHC）；C：PTEN失表达（IHC）；D：EGFR（L858R）阴性（IHC）；E：EGFR（E746-A750）阴性(IHC)；F：ALK（D5F3）阴性（IHC）；G：MET低表达（IHC）；H：ROS1阴性（IHC）；I：EGFR高表达（IHC）；J：HER2低表达（IHC）；K：p-Akt（Ser473）阳性（IHC）；L：Ki67计数大于10%小于30%（IHC）。 Lung squamous cell carcinoma case 2, the morphology and expression of proteins in PI3K pathway (×200). A: Histological morphology (HE); B: Expression of p110*β* is high (IHC); C: Expression of PTEN is loss (IHC); D: Expression of EGFR (L858R) is negative (IHC); E: Expression of EGFR (E746-A750) is negative (IHC); F: Expression of ALK (D5F3) is negative (IHC); G: Expression of MET is low (IHC); H: Expression of ROS1 is negative (IHC); I: Expression of EGFR is high (IHC); J: Expression of HER2 is low (IHC); K: Expression of p-Akt (Ser473) is positive (IHC); L: Ki67 index is more than 10% and less than 30% (IHC).

细胞系研究显示p110α和p110β不仅上游调控因子不同，下游靶点及信号通路亦存在差异，p110α活化与p-Akt的高表达具有很好的一致性，而p110β高表达的细胞p-Akt表达水平却很低^[22]^，本研究显示NSCLC肿瘤组织p110β与p-Akt的表达不相关，与之相符，提示p110β高表达发挥细胞生物学效应的途径与磷酸化Akt无关。

本研究显示NSCLC肿瘤组织p110β高表达与ki67指数呈正相关，结合文献^[23]^报道p110β高表达的胃癌生存时间短，提示p110β高表达具有预测预后较差的潜在临床意义。

PTEN是PI3K通路中最重要的拮抗因子，将PIP3去磷酸化为PIP2以对抗PI3K的作用，PTEN缺失是包括肺癌在内多种肿瘤的发病机制之一。细胞系研究发现p110β是维持PTEN缺失肿瘤细胞增殖和运动的关键^[24]^，本研究显示NSCLC肿瘤组织p110β高表达与PTEN缺失呈正相关，在组织学水平证实了上述理论。据此，有学者^[19]^提出了一个新的肿瘤分子亚型，即PTEN缺失/p110β高表达，认为该类肿瘤的发生由PTEN缺失启动，其维持和进展则依赖于p110β高表达，治疗策略上应选择特异的p110β抑制剂，该理论目前已在前列腺癌和子宫内膜癌的细胞系研究和临床试验中得到进一步的证实，p110β抑制剂能有效抑制PTEN缺失/p110β高表达肿瘤细胞的生长而p110α抑制剂则不能^[25, 26]^。

总之，p110β高表达在NSCLC是一频发事件；与PTEN缺失密切相关，是PTEN缺失肿瘤生存和进展的关键；可导致肿瘤增殖指数升高，但却与磷酸化Akt无关。*PIK3CB*基因突变不是p110β高表达的主要原因；各类RTKs异常均未显示导致癌组织p110β表达增高的能力，不仅如此，EGFR突变者p110β的表达反而低于EGFR野生型。
